# Evaluating a Targeted Antimicrobial Stewardship Program and Its Temporal Association with Resistance Trends in a Veterinary Referral Hospital †

**DOI:** 10.3390/vetsci12080743

**Published:** 2025-08-08

**Authors:** Tomoki Motegi, Rei Fukuoka, Yuzo Tsuyuki, Dai Nagakubo, Shingo Maeda, Tomohiro Yonezawa, Ryohei Nishimura, Yasuyuki Momoi

**Affiliations:** 1 Veterinary Clinical Genetics and Information, Cooperative Department of Veterinary Medicine, Faculty of Agriculture, Tokyo University of Agriculture and Technology, Tokyo 183-8509, Japan; 2 Veterinary Medical Center, The University of Tokyo, Tokyo 113-8657, Japan; arfoka@g.ecc.u-tokyo.ac.jp (R.F.); nagakubo@vmc.a.u-tokyo.ac.jp (D.N.); surgspartan@g.ecc.u-tokyo.ac.jp (R.N.); 3 Division of Clinical Laboratory, Sanritsu Zelkova Veterinary Laboratory, Tokyo 135-0011, Japan; y-tsuyuki@san-g.com; 4 Department of Veterinary Clinical Pathobiology, Graduate School of Agricultural and Life Sciences, The University of Tokyo, Tokyo 113-8657, Japan; amaeda@g.ecc.u-tokyo.ac.jp (S.M.); tomohiro.yonezawa@gmail.com (T.Y.); momoi@g.ecc.u-tokyo.ac.jp (Y.M.)

**Keywords:** antimicrobial stewardship, antibiogram, antimicrobial resistance, extended-spectrum β-lactamase, methicillin-resistant staphylococci

## Abstract

Antimicrobial resistance is an increasing concern in companion animal practice; however, effective mitigation strategies in veterinary referral hospitals remain underexplored. This study investigated whether antimicrobial stewardship interventions guided by hospital-specific resistance patterns and prescribing data could improve resistance outcomes in a secondary care veterinary setting. Using data from 2016 to 2018 at the University of Tokyo Veterinary Medical Center, a targeted intervention was developed and implemented in early 2019, and its impact was evaluated until 2024. The intervention included Gram staining-based presumptive pathogen estimation, antibiogram-guided antimicrobial selection, clinician education, and regular feedback on prescribing practices. By 2020, overall antimicrobial use had reduced by over 50%, with marked reductions in the use of carbapenems and fluoroquinolones. By 2022, the prevalence of extended-spectrum β-lactamase-producing *Escherichia coli* and *Klebsiella* spp. decreased from 53% to 24% and 78% to 7%, respectively. However, methicillin-resistant staphylococci remained prevalent in approximately 50% of cases. These findings suggest that hospital-tailored antimicrobial stewardship programs may be associated with meaningful reductions in resistance among gram-negative pathogens, although causality cannot be confirmed due to the observational study design. Moreover, additional strategies are necessary to address persistent resistance in gram-positive organisms.

## Simple Summary

Antimicrobial-resistant infections in pets, such as dogs and cats, represent a growing concern in veterinary medicine due to their complexity and associated health risks. This study explored strategies to optimize antibiotic use within a veterinary hospital. The study found that broad-spectrum antibiotics were frequently prescribed, often without a clearly justified clinical indication. In response, the hospital implemented an antimicrobial stewardship program aimed at guiding veterinarians in evidence-based antibiotic selection, incorporating laboratory diagnostics, and promoting safer prescribing practices. Following implementation, overall antibiotic use was decreased by approximately 50%, and the prevalence of multidrug-resistant bacterial strains significantly declined. These findings underscore the effectiveness of antimicrobial stewardship in reducing drug resistance, enhancing patient outcomes in veterinary care, and potentially limiting zoonotic transmission of resistant pathogens to humans.

## 1. Introduction

Antimicrobial resistance (AMR) in companion animals, particularly dogs and cats, has been steadily increasing and is now a significant concern in veterinary clinical practice. Although various antimicrobial stewardship programs (ASPs) have been proposed to mitigate this trend [1,2,3], effective strategies for reducing resistance rates in small animal practice, especially in veterinary referral hospital settings, remain underdeveloped. Inappropriate antimicrobial use is influenced by multiple factors, including institutional prescribing culture, diagnostic limitations, and case complexity, particularly in secondary or referral facilities [4,5]. Effective interventions must be context-specific and guided by data generated in each hospital environment.

Previous studies have shown that restricting the use of third-generation cephalosporins and fluoroquinolones can reduce the prevalence of methicillin-resistant staphylococci (MRS) and extended-spectrum β-lactamase (ESBL)-producing *Escherichia coli* in primary care veterinary settings [6,7]. Furthermore, cumulative antibiograms have proven useful for monitoring resistance trends and guiding antimicrobial selection [8]. They have also been recommended in national and international guidelines to improve antimicrobial use in companion animal practice [2,4,5]. However, no study has employed antibiogram-based approaches to identify hospital-specific hazard factors or determine critical control points for resistance emergence in veterinary referral hospitals. Moreover, although antimicrobial use and resistance data collected within hospitals offer valuable insights, such internal monitoring systems have not been widely leveraged to design tailored interventions [9,10,11].

We hypothesized that characterizing current antimicrobial use and resistance patterns, followed by the implementation of targeted interventions based on these findings, could effectively reduce hospital-associated AMR. Therefore, this study aimed to develop a hospital-specific cumulative antibiogram, evaluate local antimicrobial use and resistance patterns, and assess their utility in identifying key intervention points to reduce AMR in a secondary care companion animal hospital.

Antimicrobial resistance is widely recognized as a global One Health challenge, arising from interconnected factors in human, animal, and environmental health. International bodies such as the WHO, WOAH, and FAO have emphasized the importance of integrated AMR surveillance and control strategies across all sectors, including companion animal medicine [9]. Veterinary antimicrobial stewardship, particularly in referral settings, plays a vital role in reducing selective pressure and limiting the potential zoonotic transmission of resistant pathogens.

## 2. Materials and Methods

### 2.1. Analysis of Antimicrobial Use and Resistance for Identification of Critical Control Points

This study was conducted at the University of Tokyo Veterinary Medical Center, a secondary referral hospital providing advanced care for companion animals. The study focused on companion animals, predominantly dogs and cats, with antimicrobial prescriptions managed by approximately 50 full-time veterinary professionals, comprising experienced clinicians and rotating clinical interns. All prescribing activities were systematically documented through a centralized electronic medical record system, allowing for consistent data capture and longitudinal evaluation of antimicrobial usage trends. Clinical bacterial isolates and antimicrobial prescription data from August 2016 to August 2018 were retrospectively analyzed to identify hospital-specific hazard points contributing to AMR. For historical comparison, dispensing records from 2013 to 2014 were also reviewed to evaluate trends in second-line antimicrobial use. The antimicrobials analyzed in this study included agents from the standard susceptibility testing panel and those available for clinical use during the analysis period. These were categorized as follows:

− β-lactams: ampicillin (ABPC), amoxicillin (AMPC), amoxicillin-clavulanic acid (AMPC-CVA), cefazolin (CEZ), cefalexin (CEX), faropenem (FRPM), imipenem/cilastatin (IPM/CS)
− Fluoroquinolones: enrofloxacin (ERFX), orbifloxacin (OBFX)
− Aminoglycosides: amikacin (AMK)
− Macrolides: clindamycin (CLDM)
− Tetracyclines: doxycycline (DOXY), minocycline (MINO)
− Others: trimethoprim-sulfamethoxazole (ST), chloramphenicol (CP), and fosfomycin (FOM)

Bacterial identification was examined by a matrix-assisted laser desorption/ionization-time of flight mass spectrometry (MALDI Biotyper System, Bruker Corporation, Billerica, MA, USA), and then antimicrobial susceptibility testing (AST) was outsourced to the Sanritsu Zelkova Veterinary Laboratory and conducted in accordance with the Clinical and Laboratory Standards Institute (CLSI) M100-S26 or CLSI VET01-S guidelines [12,13]. MRS were identified based on phenotypic resistance to oxacillin using MIC, and ESBL production was confirmed using combination disk testing with clavulanic acid, as per the CLSI guidelines. Antimicrobial use was calculated as days of therapy [14], defined as the total number of calendar days on which each antimicrobial agent was administered to a patient, irrespective of dosage or frequency. The numerator (annual days of therapy) was obtained by summing these values across all patients within each calendar year. The denominator (patient-days) was defined as the cumulative total of daily inpatient census counts over the same period. Annual cumulative antibiograms were constructed to visualize susceptibility trends for major pathogens, particularly *Staphylococcus pseudintermedius* and the family *Enterobacteriaceae* (e.g., the genera *Escherichia* and *Klebsiella)*. These data were integrated to identify prescribing hazards contributing to antimicrobial resistance, as informed by previous studies. Prescriptions were evaluated for the following risk factors: (1) Absence of a documented diagnostic rationale, (2) omission or non-utilization of culture and AST results, (3) empirical antimicrobial choices inconsistent with laboratory findings, (4) inappropriate selection of antimicrobial spectrum, dosage, or treatment duration, and (5) excessive use of broad-spectrum agents. Communication with clients regarding antimicrobial use was not assessed due to insufficient documentation in the medical records.

### 2.2. Intervention Strategy

**Figure 1:**
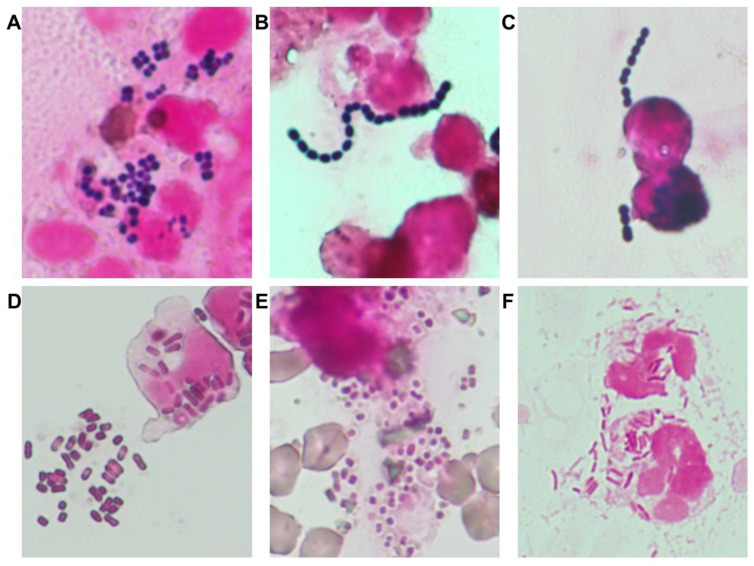
Representative Gram-staining images of clinically relevant bacterial pathogens in companion animals. gram-positive cocci: (**A**) *Staphylococcus* spp.—These bacteria typically appear in grape-like clusters due to symmetrical division in two perpendicular planes; often forming tetrads. This characteristic arrangement serves as a distinguishing feature that aids in the visual identification of *Staphylococcus* species. (**B**) *Enterococcus* spp.—Usually observed in short chains or pairs; these cocci are oval or slightly elongated. Chains generally contain fewer than seven cocci. *Enterococcus* species are notable for their intrinsic resistance to cephalosporins. (**C**) *Streptococcus* spp.—Appear as long; uniform chains, typically with seven or more cocci per chain. Penicillin-class antimicrobials remain the first-line treatment for infections caused by *Streptococcus* spp. gram-negative rods. (**D**) *E. coli*—These rods typically have a width-to-length ratio ranging from 1:2 to 1:3. (**E**) *Klebsiella* spp.—Generally thicker than *E. coli*, they may possess a visible capsule; resulting in a blurred or halo-like outline under the microscope. (**F**) *Pseudomonas* spp.—These organisms appear as long; narrow; and sometimes curved rods; often displaying uneven staining. They exhibit intrinsic resistance to many antimicrobials, and fluoroquinolones are commonly used as first-line agents.

Based on the analysis of the 2016–2018 data, several concerns regarding antimicrobial use were identified, consistent with findings from previous studies [4,5]. These included empiric prescriptions without pathogen identification, a lack of effective second-line options for multidrug-resistant organisms, excessive use of carbapenems and fluoroquinolones, and misinterpretation of AST results due to reporting on intrinsically resistant organisms. In response, a hospital-specific antimicrobial stewardship intervention was initiated in January 2019. All strategies were implemented as non-mandatory recommendations without enforcement authority. The core components included:

1. Proactive use of Gram staining:

Gram staining was implemented as a frontline diagnostic tool to guide early pathogen estimation, particularly for six major organisms relevant to small animal medicine (Figure 1), as supported by prior studies [15,16,17]. Gram staining results were used in conjunction with antibiogram trends and clinical severity to guide empirical therapy.

2. Empirical therapy optimization:

Empirical treatment protocols were revised based on Gram stain results and annual antibiogram data to identify and eliminate ineffective antibiotic choices. Narrow-spectrum agents were prioritized, and the use of broad-spectrum antibiotics was discouraged unless supported by microbiological evidence. Additionally, pharmacokinetic/pharmacodynamic (PK/PD)-based dosing regimens have been developed for certain antibiotics using Monte Carlo simulations [18].

3. Restriction of high-risk antimicrobials:

Use of high-risk agents, particularly carbapenems and fluoroquinolones, was restricted to cases supported by culture and sensitivity data.

4. Refinement of AST result reporting:

Laboratory reporting protocols were modified to suppress susceptibility data for organisms with known intrinsic resistance, thereby preventing clinicians from misinterpreting AST reports and inadvertently prescribing ineffective agents.

Key stewardship practices were primarily delivered through direct clinical consultations, including bedside discussions with attending clinicians, referrals from the intensive care unit, and targeted email communication. These methods enabled real-time, individualized feedback and were particularly effective for supporting decision-making in complex or high-risk cases. Educational components were delivered to all clinical staff, including rotating interns, through structured seminars and case-based discussions. Topics included principles of empirical and definitive antimicrobial selection, interpretation of antibiograms, pharmacokinetic/pharmacodynamic (PK/PD) concepts, and case audits. The intervention’s effectiveness was evaluated by comparing antimicrobial use patterns and resistance rates before and after the intervention.

### 2.3. Statistical Analysis

Bayesian inference was used to identify antimicrobial classes that were significantly overprescribed relative to national trends. The higher of the 2016 or 2020 national usage proportions (based on official sales data from the National Veterinary Assay Laboratory: https://www.maff.go.jp/nval/yakuzai/yakuzai_p3_6.html: accessed on 24 June 2025) was used as the prior. Institutional prescription counts were used to update the beta distribution, and posterior distributions were generated by drawing 10,000 samples using the Rbeta function in R. Overprescription was considered statistically significant if the posterior probability was >0.95.

To assess resistance trends from 2017 to 2024, binomial logistic regression was performed with year as a continuous predictor. The log-odds slope and corresponding odds ratio (OR) per year were calculated to evaluate significant changes in resistance rates. While *Streptococcus* spp., *Enterococcus* spp., and *Pseudomonas* spp. were initially included in the antibiogram framework, they were excluded from resistance trend analysis after 2018 due to consistently low isolate counts, falling below the 30-isolate threshold recommended by CLSI for annual cumulative antibiograms [19]. All analyses were performed in R (v4.4.1) using the ggplot2 (v3.5.1), dplyr (v1.1.4), tidyr (v1.3.1), and multcomp (v1.4.26) packages.

## 3. Results

### 3.1. Bacterial Epidemiology, Antimicrobial Resistance, and Identification of Critical Control Points

Between August 2016 and August 2018, 878 clinical bacterial isolates were collected from the University of Tokyo Veterinary Medical Center. The six most frequently identified organisms were *Staphylococcus* spp. (n = 214), *E. coli* (n = 185), *Enterococcus* spp. (n = 125), *Klebsiella* spp. (n = 96), *Streptococcus* spp. (n = 56), and *Pseudomonas* spp. (n = 45) (Figure 1). Among these, antimicrobial resistance was notably prevalent: MRS accounted for 64% of *Staphylococcus* spp. isolates; ESBL-producing *E. coli* and *Klebsiella* spp. were detected in 51% and 78% of respective isolates.

Bayesian analysis revealed that as early as 2013, the institutional use of fluoroquinolones and carbapenems significantly exceeded the national usage proportions, with posterior probabilities surpassing 0.99. This trend persisted through 2016, during which additional overprescription of penems was also identified (posterior probability = 1.00), indicating sustained overuse of multiple broad-spectrum agents. Based on the analysis of antibiotics prescribed, several hazards related to prescribing were identified. Among the 11,184 prescriptions reviewed, 3318 (29.7%) lacked any documented diagnostic justification. Of the 878 culture-positive cases, 92 prescriptions (10.5%) involved antimicrobial agents that were inconsistent with the corresponding AST results. In 10 patients, empiric administration of extremely broad-spectrum antimicrobials (e.g., carbapenems or aminoglycosides) was required from the outset due to the absence of viable second-line options. Furthermore, in surgical procedures, the use of antimicrobials at the time of discharge varied among clinicians, with a more than five-fold difference in prescribing frequency. Integrating AST results with dispensing data identified several potential critical control points, including the empirical overuse of second-line agents, insufficient prioritization of narrow-spectrum alternatives, and inconsistent antimicrobial selection based on Gram-staining results and clinical severity.

### 3.2. Impact of Intervention on Antimicrobial Use and Resistance Trends

**Figure 2:**
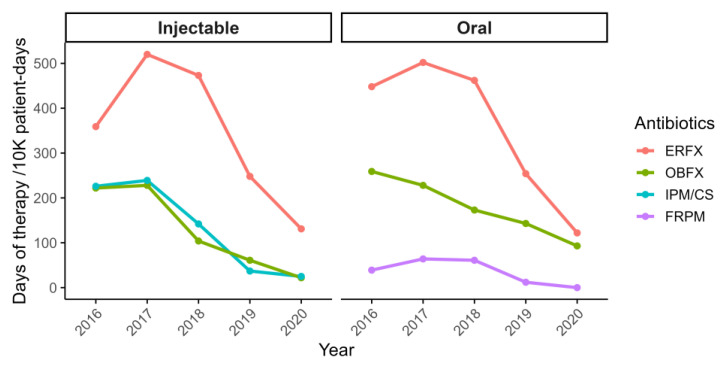
Trends in antimicrobial prescriptions for companion animals from 2016 to 2020. Antimicrobial prescription trends for companion animals between 2016 and 2020 are expressed as days of therapy per 10,000 patient-days. The data are categorized by route of administration, with oral agents shown on the left and injectable agents on the right. OBFX: orbifloxacin; ERFX: enrofloxacin; FRPM: faropenem; IPM/CS: imipenem/cilastatin.

Following the implementation of antimicrobial stewardship interventions, prescription rates documented in medical records decreased by over 50% by 2020, with total days of therapy per 10,000 patient-days declining from 5262 to 2575. Notably, usage of injectable antimicrobials showed marked reductions: imipenem/cilastatin decreased by 88%, enrofloxacin (ERFX) by 71%, and orbifloxacin (OBFX) by 88%. Oral formulations followed similar trends, with ERFX and OBFX decreasing by 72% and 90%, respectively, and faropenem was completely discontinued (100% reduction; Figure 2). Additionally, we assessed total antimicrobial use across all drug classes. As shown in Supplementary Table S1, the reduction in days of therapy was observed broadly, including among first-line antimicrobials such as aminopenicillins and cephalosporins.

Corresponding shifts in resistance rates were also observed, particularly among gram-negative bacteria. By 2022, the prevalence of ESBL-producing *E. coli* decreased from 53% to 24%, while that of ESBL-producing *Klebsiella* spp. dropped from 78% to 7%. In contrast, MRS prevalence remained high, showing only a modest decline (from 64% to 53%). To assess these trends, binomial logistic regression was performed using data from 2017 to 2022. A statistically significant decreasing trend in resistance was observed for all three pathogens. The OR per year was 0.90 (*p* = 0.0179) for MRS, 0.78 (*p* = 2.00 × 10^−6^) for ESBL-producing *E. coli*, and 0.55 (*p* = 9.38 × 10^−11^) for ESBL-producing *Klebsiella* spp.

**Figure 3:**
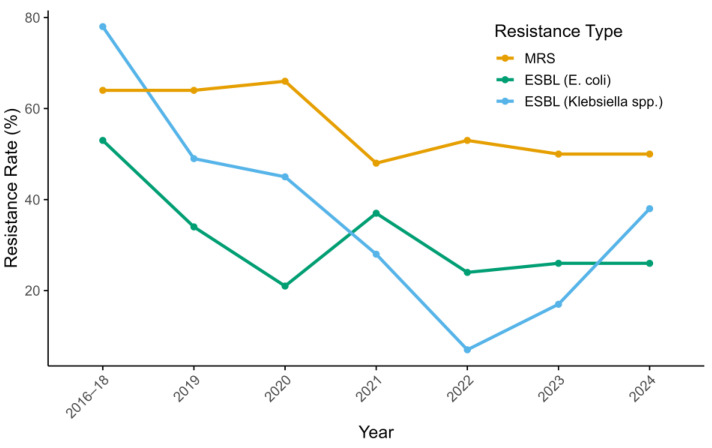
Trends in antimicrobial resistance rates among companion animal isolates from 2016 to 2024. The analysis presents annual proportions of antimicrobial-resistant isolates identified through clinical microbiological testing of companion animals. To evaluate the annual trends in antimicrobial resistance, binomial logistic regression was performed using the year as a continuous predictor from 2017 to 2022. MRS showed a modest but statistically significant decline in resistance rates over time (odds ratio [OR] per year = 0.90, *p* = 0.0179). In contrast, more pronounced reductions were observed for ESBL-producing *E. coli* (OR = 0.78, *p* = 2.00 × 10^−6^) and ESBL-producing *Klebsiella* spp. (OR = 0.55, *p* = 9.38 × 10^−11^). MRS: methicillin-resistant staphylococci; ESBL: extended-spectrum β-lactamase.

In September 2022, stewardship leadership transitioned from the original lead (TM) to a successor (RF). During 2023–2024, MRS rates remained stable at 50%, the prevalence of ESBL-producing *E. coli* remained at 26%, and ESBL-producing *Klebsiella* spp. increased from 17% to 38% (Figure 3). To assess the potential effect of leadership change on resistance patterns, a separate logistic regression was used to compare resistance rates between the two-year periods before (2021–2022) and after (2023–2024) the transition. The ORs for the earlier period relative to the latter were 1.02 (*p* = 0.918) for MRS, 1.31 (*p* = 0.310) for ESBL-producing *E. coli*, and 0.70 (*p* = 0.498) for ESBL-producing *Klebsiella* spp., indicating no statistically significant impact of the leadership transition on resistance trends.

## 4. Discussion

The antimicrobial stewardship intervention implemented at our secondary referral hospital led to a substantial reduction in overall antimicrobial use and coincided with marked improvements in resistance rates among gram-negative bacteria. By 2020, total antimicrobial prescriptions had decreased by over 50%, with the most significant reductions observed in broad-spectrum agents such as carbapenems and fluoroquinolones. These reductions were temporally associated with declines in the prevalence of ESBL-producing *E. coli* and *Klebsiella* spp., which decreased from 53% to 24% and from 78% to 7%, respectively, by 2022. These findings are consistent with previous studies highlighting the role of β-lactam and fluoroquinolone restriction in mitigating resistance among Enterobacteriaceae [6,20]. Similar reductions in ESBL prevalence have been reported following the implementation of ASP in human clinics [21,22], and the strategy of antibiotic restriction reinforces the clinical utility of cumulative antibiograms for empirical treatment selection. These findings also underscore the importance of implementing antimicrobial stewardship programs in companion animal hospitals as part of the broader One Health strategy for combating antimicrobial resistance, as advocated by the WHO, WOAH, and FAO [9].

In contrast, the prevalence of MRS declined only modestly, from 64% to 53% by 2022, and remained stable at approximately 50% thereafter. This limited response suggests that our stewardship strategies were less effective in controlling resistance among MRS. Although previous studies have reported reductions in MRS rates following antimicrobial stewardship efforts [6,8], several factors may have limited effectiveness in this study. These include suboptimal adherence to stewardship recommendations [23,24], continued empirical use of β-lactam antibiotics for presumed staphylococcal infections, and insufficient de-escalation based on culture results [25,26]. Especially, the interventions implemented in this study were non-compulsory and based solely on recommendations, which may have limited their impact. Additionally, unlike gram-negative organisms, which are primarily driven by antimicrobial selective pressure, MRS prevalence may not be as responsive to stewardship interventions alone [27]. One reason is to suspect the presence of different resistance mechanisms and ecological persistence [28,29,30,31]. Therefore, additional strategies such as enhanced infection control measures may be required to achieve further reductions.

Although stewardship leadership transitioned in September 2022 from the original lead (TM) to a new clinician (RF), statistical analysis revealed no significant changes in resistance rates across the 2021–2022 and 2023–2024 periods. ORs comparing the earlier to the latter period were 1.02 for MRS, 1.31 for ESBL-producing *E. coli*, and 0.70 for ESBL-producing *Klebsiella* spp. However, the increase in ESBL-producing *Klebsiella* from 17% to 38% during the latter period suggests a potential weakening of stewardship impact, warranting further attention. These findings underscore the importance of continuity in program leadership, consistent institutional support, and structured handovers to maintain long-term effectiveness. Periodic reviews of antibiogram trends are also essential to adapt interventions to evolving resistance dynamics.

This study has several limitations. First, the adoption rate of individual recommendations was not assessed due to the voluntary nature of the interventions. Nevertheless, institution-wide prescribing and resistance data were used to determine the overall effect. Second, the distinct effects of each intervention component were not analyzed separately, as they were implemented as part of a coordinated strategy. Third, external infection control practices associated with the COVID-19 pandemic, such as increased handwashing, masking, and environmental sanitation, may have influenced resistance trends. However, the use of multi-year pre- and post-intervention data helped mitigate this limitation and supported the internal validity of the findings. Moreover, although the temporal concordance between reduced antimicrobial use and improved AMR patterns is noteworthy, the observational nature of this study precludes definitive causal inference. It remains plausible that referred patients introduced resistant organisms, and the absence of molecular epidemiological analyses limits our ability to differentiate in-hospital transmission from external acquisition. Additionally, because bacterial isolates were typically obtained at the time of referral, before in-hospital treatment, the observed resistance patterns may reflect antimicrobial exposure in referring facilities. However, it is possible that the stewardship practices at our institution, including case-based consultations and ongoing communication with primary care veterinarians, contributed indirectly to more judicious antimicrobial use in referring clinics. Furthermore, some patients were referred multiple times for chronic or recurrent infections, potentially allowing the effects of stewardship interventions to accumulate across successive encounters. These factors remain speculative but may have influenced the observed resistance trends. Accordingly, the associations observed herein should be interpreted with caution, as they are hypothesis-generating rather than conclusive evidence of the efficacy of antimicrobial stewardship interventions.

## 5. Conclusions

Hospital-specific antimicrobial stewardship programs, including in-hospital education and antibiotic selection guided by antibiograms, were associated with substantial reductions in antimicrobial use and resistance rates among gram-negative pathogens, notably a marked decline in ESBL-producing *E. coli* and *Klebsiella* spp. However, the limited and inconsistent reduction in MRS highlights the need for enhanced implementation fidelity and complementary infection control measures. Sustained program leadership, periodic resistance surveillance, and structured engagement with referring clinics may further enhance the long-term effectiveness of stewardship efforts across bacterial species.

## Data Availability

The data presented in this study are available on request from the corresponding author due to institutional policy and the inclusion of clinical records that may contain sensitive or indirectly identifiable information.

## Supplementary Materials

The following supporting information can be downloaded at: https://www.mdpi.com/article/10.3390/vetsci12080743/s1, Table S1: Annual antimicrobial use (days of therapy per 10,000 patient-days) by drug class from 2016 to 2020.

## Abbreviations

The following abbreviations are used in this manuscript:

ABPC	Ampicillin
AMK	Amikacin
AMPC	amoxicillin
AMR	Antimicrobial Resistance
ASP	Antimicrobial Stewardship Program
AST	Antimicrobial Susceptibility Testing
CEX	Cefalexin
CEZ	Cefazolin
CLDM	Clindamycin
CLSI	Clinical and Laboratory Standards Institute
CP	Chloramphenicol
CVA	Clavulanic acid
DOXY	Doxycycline
ERFX	Enrofloxacin
ESBL	Extended-Spectrum β-Lactamase
FOM	Fosfomycin
FRPM	Faropenem
IMP/CS	Imipenem/cilastatin
MINO	Minocycline
MRS	Methicillin-Resistant Staphylococci
OBFX	Orbifloxacin
OR	Odds Ratio
PK/PD	Pharmacokinetic/Pharmacodynamic
ST	Sulfamethoxazole-trimethoprim
